# Use of a Text Message-Based Pharmacovigilance Tool in Cambodia: Pilot Study

**DOI:** 10.2196/jmir.2477

**Published:** 2013-04-16

**Authors:** Sophie Baron, Flavie Goutard, Kunthy Nguon, Arnaud Tarantola

**Affiliations:** ^1^Epidemiology and Public Health Unit (epi@ipc)Institut Pasteur du CambodgePhnom PenhCambodia; ^2^AGIRs unitCentre de coopération internationale en recherche agronomique pour le développement (CIRAD)MontpellierFrance

**Keywords:** cellular phone, text messages, texting, short message service, vaccines, adverse events, surveillance, adverse drug reaction reporting systems, pharmacovigilance

## Abstract

**Background:**

There is no functional pharmacovigilance system in Cambodia to our knowledge. Mobile phone–based tools, such as short message service (SMS) text messages, are increasingly used for surveillance purposes.

**Objective:**

To pilot-test the FrontlineSMS mobile phone–based tool for notification of adverse events, using Cambodia’s only International Vaccination Center at the Institut Pasteur du Cambodge as a field site.

**Methods:**

People receiving vaccinations, aged over 18 years, and who owned a cell phone were recruited in the study following informed consent. The names and mobile phone numbers of the participants interviewed were entered each day into the FrontlineSMS software. Two days after being vaccinated, participants received an automatically generated SMS text message asking whether any adverse events had occurred. Their SMS reply was number-coded and exported from the software daily to an Excel spreadsheet and examined before being saved. If the participant replied with a code for a severe adverse event (8 or 9), they were automatically advised to consult the nearest doctor.

**Results:**

The active surveillance study was conducted over 72 days in the spring of 2012. Patients agreed to be asked by SMS text message whether unwanted events had occurred after vaccination. Of 1331 persons aged over 18 years referred to the vaccination unit, 184 (13.8%) were asked and agreed to participate. When texted for clinical status 48 hours after vaccination, 52 (28.3%) participants did not reply, 101 (54.9%) sent an immediate SMS reply, and 31 (16.8%) sent an SMS reply after additional prompting. Of the initial 184 participants, 132 (71.7%) replied. These 132 participants received 135 vaccine doses and 109 (82.6%) reported no adverse events, whereas 23 (17.4%) reported adverse events, all benign.

**Conclusions:**

Notification using an SMS-based text message system is already used in Cambodia for syndromic surveillance in health centers and reporting by health care workers. Our results show that such tools can also be useful for notification by patients or health users in Cambodia, especially in an urban setting.

## Introduction

The burden of disease can be threefold in developing countries faced with communicable diseases, noncommunicable diseases, and sociobehavioral illnesses [1]. Most of these may require treatment or prevention through medication or vaccines. In addition to the expectable adverse events in the normal usage of registered drugs, developing countries also face a plague of counterfeit or substandard drugs [2-5]. Pharmacovigilance—a form of epidemiological surveillance that monitors the occurrence of adverse events of drugs [6,7] or vaccines [8-10] to guide timely corrective action and mitigate risk—is an essential tool for patient safety in developed countries and in an increasing number of developing countries [11]. Cambodia, a developing country in Southeast Asia, is not among the 109 countries participating in the World Health Organization (WHO) Programme for International Drug Monitoring maintained in collaboration with the Uppsala Monitoring Center in Uppsala, Sweden [12]. To our knowledge, there is no functional pharmacovigilance program in Cambodia.

Pharmacovigilance has been conducted using complex and rigorous notification procedures in developed countries, but it faces challenges in developing countries because of issues with clinician awareness, clinical expertise, or nonfunctional reporting systems [11,13,14]. Therefore, some programs or countries have circumvented these challenges by implementing mobile phone-based and Web-based tools using short message service (SMS) text messaging for immediate notification of adverse events [15,16].

**Figure 1:**
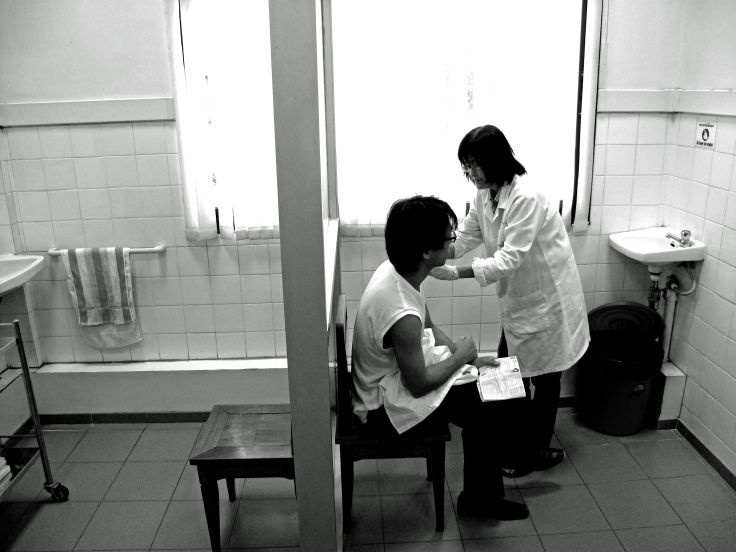
Vaccination at the International Vaccination Center, Institut Pasteur du Cambodge.

**Figure 2:**
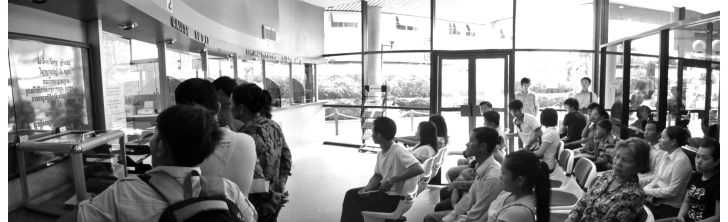
The waiting room of the international vaccination center at the Institut Pasteur du Cambodge.

FrontlineSMS is one such reporting tool [17]. It has been used in Cambodia for various health-related projects, but always for information exchange among health providers and stakeholders [18-20]. An Epidemiology Master student helped pilot its use for surveillance in direct link with health users in a vaccination center. More than 25,000 vaccine doses were administered to 16,630 health users in 2012 (Figure 1) referred to the International Vaccination Center at the Institut Pasteur du Cambodge (IPC), the only such vaccination center in Cambodia (Figure 2). The aim of this study was to field-test the FrontlineSMS software to see whether it could provide effective and timely notification of vaccine adverse events.

## Method

The research project received ethical approval from the national ethics committee on health research on February 17, 2012. Data collection began March 12, 2012, and ended May 31, 2012.

During that period, a research assistant from the epidemiology and public health unit at IPC (a native Khmer speaker) spent several hours each day at the International Vaccination Center to recruit participants. Participants were eligible to be included in the study if they were aged over 18 years, came to the center to be vaccinated themselves (ie, not for their children), agreed to participate, owned a cell phone, and knew how to send SMS text messages. If so, the research assistant informed them about the study, read through the protocol, explained the objectives of the pharmacovigilance project in Khmer, and asked the participants to complete an informed consent form. Information about their name, age, place of residence, type of vaccine, and mobile phone service provider were collected. The names and mobile phone numbers of the participants interviewed were entered each day in the FrontlineSMS software.

Two days after being vaccinated, participants received an automatically generated SMS text message. This message thanked them for participating and asked whether any adverse events had occurred. Their SMS reply was number-coded as follows: 0=no adverse event, 1=mainly redness and/or pain at the injection site, 2=mainly fatigue and/or weakness, 3=mainly headaches, 4=mainly fever, 5=mainly runny or congested nose, 6=mainly muscle pains, 7=mainly abdominal pain, 8=seizures or neurological problems, and 9=severe allergic reaction. Only 1 code was allowed per reply. Messages received were exported from the software daily to an Excel spreadsheet and examined before being saved.

Upon receiving the text reply, a software-generated message was sent. If the codes corresponded to no or a moderate adverse event (codes 0-7), the reply was: “We have received your message. Thank you for having participated.” If the participant sent back a code for a severe adverse event (8 or 9), the reply read: “You have reported a severe adverse event. Please consult the nearest doctor as soon as possible. If in Phnom Penh, we recommend that you refer to Hôpital Calmette.” (The Hôpital Calmette is a national reference hospital where the emergency department team had been informed of the study.) In cases when a severe adverse event was reported, the research assistant called the participant’s cell phone number directly to follow-up on the participant. If the case was referred to Hôpital Calmette, transportation costs were covered by the study. There was no other financial compensation to participants, including for costs associated with sending SMS text messages.

## Results

**Figure 3:**
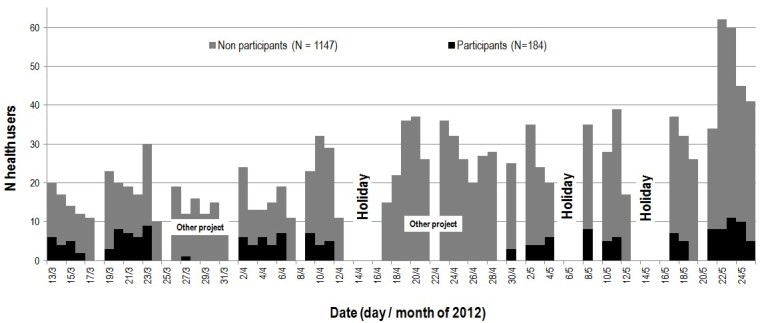
Number of health users at the international vaccination center and inclusions in the SMS pilot study, Institut Pasteur du Cambodge, March-May 2012.

The study took place between March 13 and May 25, 2012, for a total of 72 days: 53 days of which the International Vaccination Center was open and 19 days of which it was closed (weekend or national holidays, Figure 3). During some of those days, the research assistant did not recruit participants because she was involved in another study.

**Figure 4:**
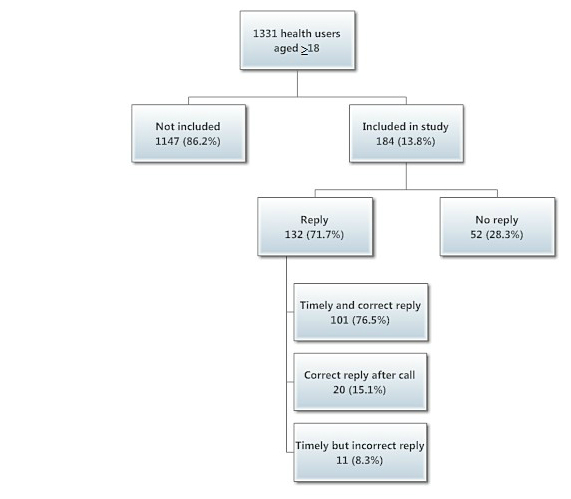
Flowchart of patient recruitment in the pilot SMS text-based pharmacovigilance study at International Vaccination Center, Institut Pasteur du Cambodge, March-May 2012 (percentage total may not equal 100% due to rounding).

During the 53 days that the International Vaccination Center was open in that period, 1331 persons aged over 18 years (684 women and 647 men) came for vaccinations at the center (mean 25.1 persons aged ≥18 years per day, Figure 4).

Of the 1331 vaccinees, 184 were asked and agreed to participate to this pilot study (97 female, 87 male). Of these 184 participants, 165 (90.2%) resided in Phnom Penh; 6 (3.3%) in Kampong Speu; 3 (1.6%) each in Kandal and Kompong Cham, respectively; 2 (1.1%) in Siem Reap; and 1 (0.5%) each in Takeo, Battambang, Mondulkiri, and Rattanakiri, respectively. Mean age of all 1331 health users during that period was 34.8 years (SD 12.7, range 18-95), although the subgroup of study participants was younger (mean 26.9 years, SD 7.8, range 18-65). The 184 participants subscribed to 6 cell phone companies, the largest of which accounted for 83 (45.1%) of the participants.

The 184 health users who initially agreed to participate in the study received a total of 192 vaccine doses for 17 different diseases (Table 1).

**Table 1:** Vaccines administered to study participants and reported adverse events for the pharmacovigilance pilot study at the Institut Pasteur du Cambodge from March to May 2012 (N=184).

Vaccine^a^	No adverse event	Mainly redness and/or pain at the injection site	Mainly fatigue and/or weakness	Mainly fever	Mainly runny or congested nose	No reply	Total
Hepatitis A	1	0	0	0	0	0	1
Hepatitis B	39	4	3	1	0	30	77
Haemophilus influenza	2	0	0	1	0	0	3
Japanese encephalitis	9	0	0	0	0	1	10
MMR	0	0	0	0	0	1	1
Meningitis	2	0	0	0	0	0	2
Chickenpox	1	0	0	0	0	0	1
Pneumococcus	2	0	0	0	0	2	4
Rubella	0	0	0	0	0	1	1
Tetanus	13	3	1	0	0	3	20
Tetanus & rabies	2	0	0	0	0	2	4
DTCP	2	1	1	0	1	0	5
Typhoid	2	0	0	0	0	0	2
HPV	10	3	0	1	2	4	20
Influenza	17	0	0	0	0	5	22
Influenza & Hib	2	0	0	0	0	0	2
Influenza & tetanus	1	0	0	0	0	0	1
Rabies	0	1	0	0	0	0	1
Yellow fever	4	0	0	0	0	2	6
Yellow fever & meningitis	0	0	0	0	0	1	1
Total	109	12	5	3	3	52	184

^a^ MMR: measles, mumps, rubella; DTCP: diphtheria, tetanus, pertussis, polio; HPV: human papillomavirus; Hib: *Haemophilus influenzae* serotype B.

Most of the vaccinations given were against hepatitis B (41.8%), influenza (11.9%), tetanus (10.9%), and human papillomavirus (HPV) (10.9%). Fifty-two participants (28.2%) did not send a reply (including 30 of 77 who received vaccination against hepatitis B), and 132 (71.7%) did send a reply, of which 101 (76.5%) completed the study as per the study protocol and sent a correct SMS text reply, whereas 31 (23.5%) participants had to be contacted twice because they replied incorrectly or did not reply at all (Figure 2). These 132 respondents received a total of 137 doses of vaccine. In all, 109 (82.6%) respondents reported no occurrences of adverse events, whereas 23 (17.4%) did report adverse events, none of which were severe (Table 1). Twelve (52.2%) of these adverse events pertained to redness at the injection site, and 3 (13.0%) pertained to fever. The time between the initial sending of the SMS text and the reply from participants was documented in 120 messages (mean 0.4 days, SD 0.82, range 0-5). The reply was received within the same day in 91 (75.8%) of documented answers.

## Discussion

The objective of this pilot study was to assess the feasibility of an SMS text-based reporting tool for pharmacovigilance in Cambodia. According to the International Communications Union database, there were 69.9 mobile-cellular telephone subscriptions per 100 (%) inhabitants in Cambodia in 2011 (slightly less than India), up from 7.95% in 2005 [21]. This translates to approximately 10 million cellular telephone subscriptions in Cambodia in 2011, a country with an estimated population of approximately 14 million [22].

To our knowledge, this pilot SMS text-based system for active detection of adverse events following vaccination is the first functional pharmacovigilance system in Cambodia, the first SMS text-based surveillance system relying on health users’ participation in Cambodia, and the first to use SMS text-based surveillance for adverse event detection anywhere [20]. In this project, operating costs were modest, with only part-time activity dedicated to entering cell phone numbers, downloading SMS replies, and checking that no SMS texts received alerted to severe adverse events.

During the 2.5-month pilot phase, the participation rate was high with a 71.7% response rate. Of 77 persons who were vaccinated against hepatitis B, 30 (39%) sent no reply. Although this percentage may seem high, the hepatitis B vaccines were the most frequently administered, and the small study group makes nonreply percentages vary greatly.

With 132 participants replying and 24 reporting adverse events (none severe), the prevalence of adverse events was slightly higher than expected. The expected rate of adverse events following immunization is estimated to be in the order of 10%, except for diphtheria, tetanus, pertussis (DTP) or tetanus boosters in which fever occurs in nearly half of recipients [23]. In 2008, in Australia, 1542 adverse events (7.2 per 100,000 population) following immunization were notified by manufacturers, health professionals, or the public [24]. This passive surveillance system also includes children, who are the main recipients of vaccines. Of these adverse events, 41% were site reactions and 16% were fevers. Our data, albeit on a far more modest scale and not including children, showed comparable percentages (52.2% and 13.0%, respectively). A study conducted in the United Kingdom showed that patients tend to report more benign adverse drug reactions than health care providers, and concluded that patient-based pharmacovigilance may usefully complement health care provider reporting of adverse events [25].

The participants’ response rate was unexpectedly high, probably enhanced by the use of short and simple SMS text-based reply codes. This research project also had a research assistant to explain the protocol at length in the national language, which would not be the case in a daily operational setting where a simple leaflet or poster would provide information to the health users.

Our study suffers from biases and limitations. Firstly, the number of participants was very limited. The epidemiological findings of such a small and short study on adverse events are difficult to extrapolate. Secondly, the patients recruited all attended the IPC’s International Vaccination Center in Phnom Penh. Costs there may be higher because of state-of-the-art quality control carried out on vaccines imported primarily from Europe. Therefore, recruitment may have been biased toward younger, more affluent, well-informed, urban residents more accustomed to sending SMS text messages. This limits the possibility of extending similar systems outside of large urban centers within Cambodia, at least in the short term. Experience with SMS text-based surveillance in the rural setting found that many farmers did not know how to send text messages [18]. Thirdly, only the last-generation cellular telephones support Khmer fonts or pictures of Khmer-language text, whereas the vast majority of cellular phones do not. Lastly, some operators mainly offer voice-based communications and no data or SMS text transfers because telephone communications are relatively cheap and there is no advantage to sending a text message.

Bearing these limitations in mind, this small pilot study serves as a proof of concept that health user–sent, SMS text-based surveillance strategies can be used in an urban Cambodian setting. This is an important step in Cambodia where health surveillance systems facing numerous challenges may often be dysfunctional and where pharmacovigilance is absent. Our secondary objective was also met, which was to become proficient in the use of FrontlineSMS for other potential applications. Technology for mobile telephone–based active surveillance appears to be cheap, easy to implement, simple, and quick to be mastered by field staff. This approach will be used by the Epidemiology and Public Health Unit at the Institut Pasteur du Cambodge to implement follow-up programs, such as monitoring outcomes in rabies postexposure prophylaxis at IPC’s Rabies Prevention Clinic (over 20,300 referrals in 2011) or in prospective studies on dengue in urban settings.

## Abbreviations

DTP: diphtheria, tetanus, polio
DTCP: diphtheria, tetanus, pertussis, polio
HPV: human papillomavirus
IPC: Institut Pasteur du Cambodge
SMS: short message service
WHO: World Health Organization
